# Supplementary material for: Interconnected subsets of memory follicular helper T cells have different effector functions
Source: Nat Commun. 2017 Oct 10;8:847. doi: 10.1038/s41467-017-00843-7 (PMC5635037; doi:10.1038/s41467-017-00843-7)
Supplement: Supplementary file 1 — Supplementary information [file 41467_2017_843_MOESM1_ESM.pdf]

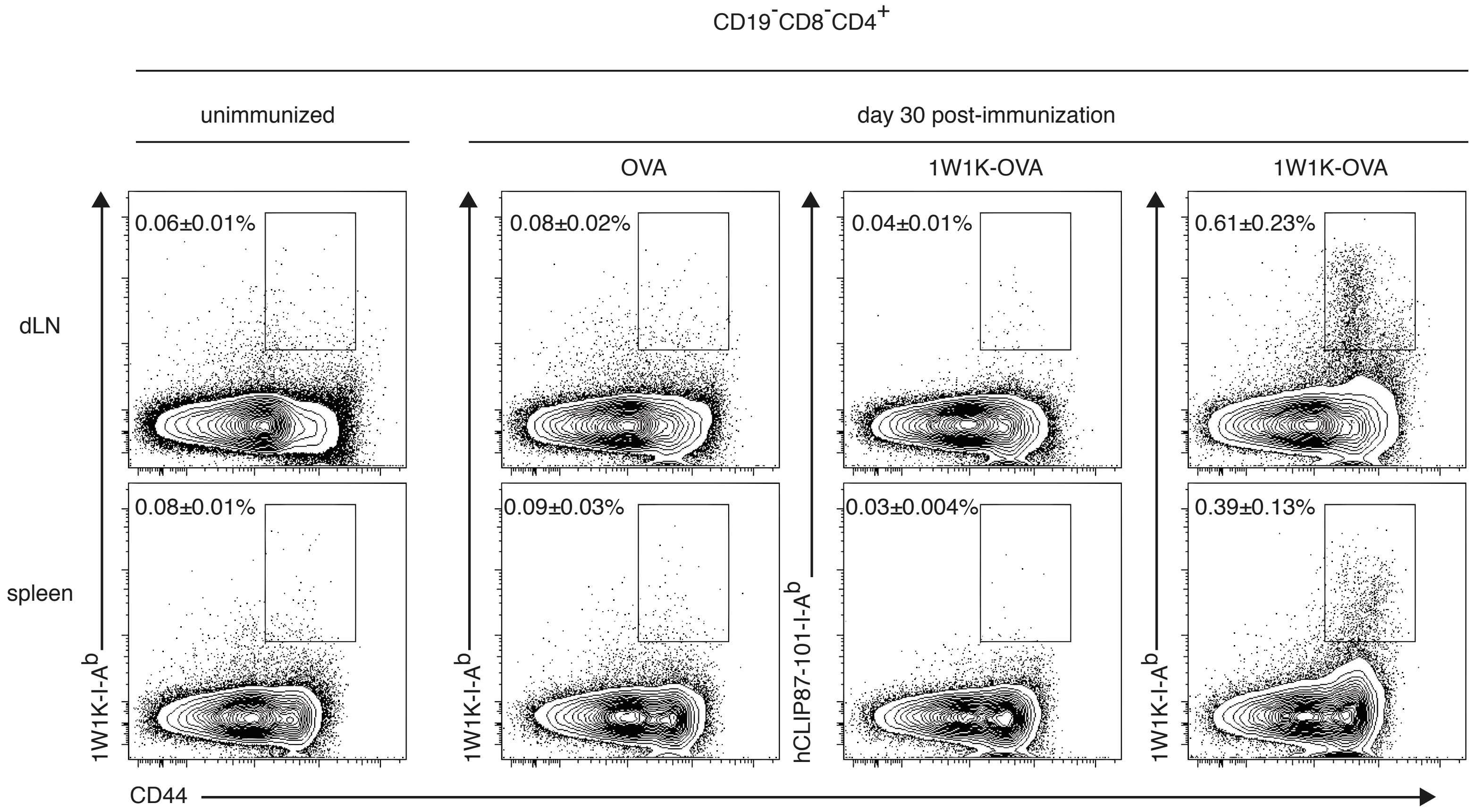

Supplementary Figure 1

Gating of 1W1K-specific Th cells in dLN and in spleen using 1W1K-I-Ab tetramer

C57BL/6 mice were immunized sc with 100 μg OVA or 1W1K-OVA in IFA+CpG.

30 days after, dLN and spleen from immunized mice or from naive mice were collected and were analyzed for the detection of 1W1K-IAb^{+}CD44^{+} and hCLIP87-101-IAb^{+}CD44^{+} Th cells.

Frequency denotes the mean value of groups and SEM (n=5/group).

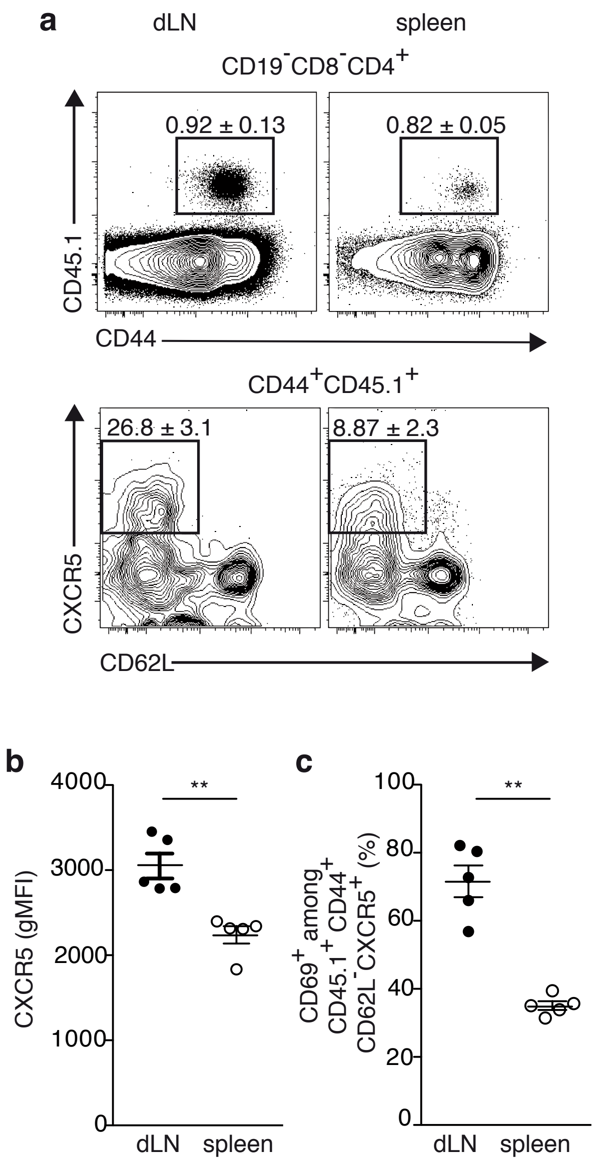

Supplementary Figure 2

Local and circulating OVA-specific memory Tfh after adoptive transfer of naïve OT-II cells.

C57BL/6 mice were transferred iv with 100000 purified $CD4^{+}CD45.1^{+}$ naïve OT-II cells and sc immunized 24h later with OVA in IFA/CpG. 30 days after, dLN and spleen were analyzed for the detection of OVA-specific Th ($CD45.1^{+}CD44^{+}$) and OVA-specific Tfh ($CXCR5^{+}CD62L^{-}$) (**a**) (mean±SEM, n=5). CXCR5 expression at the surface of OVA-specific mTfh (**b**) and frequency of $CD69^{+}$ in OVA-specific Tfh (**c**). Mann-Whitney test, **P<0.01

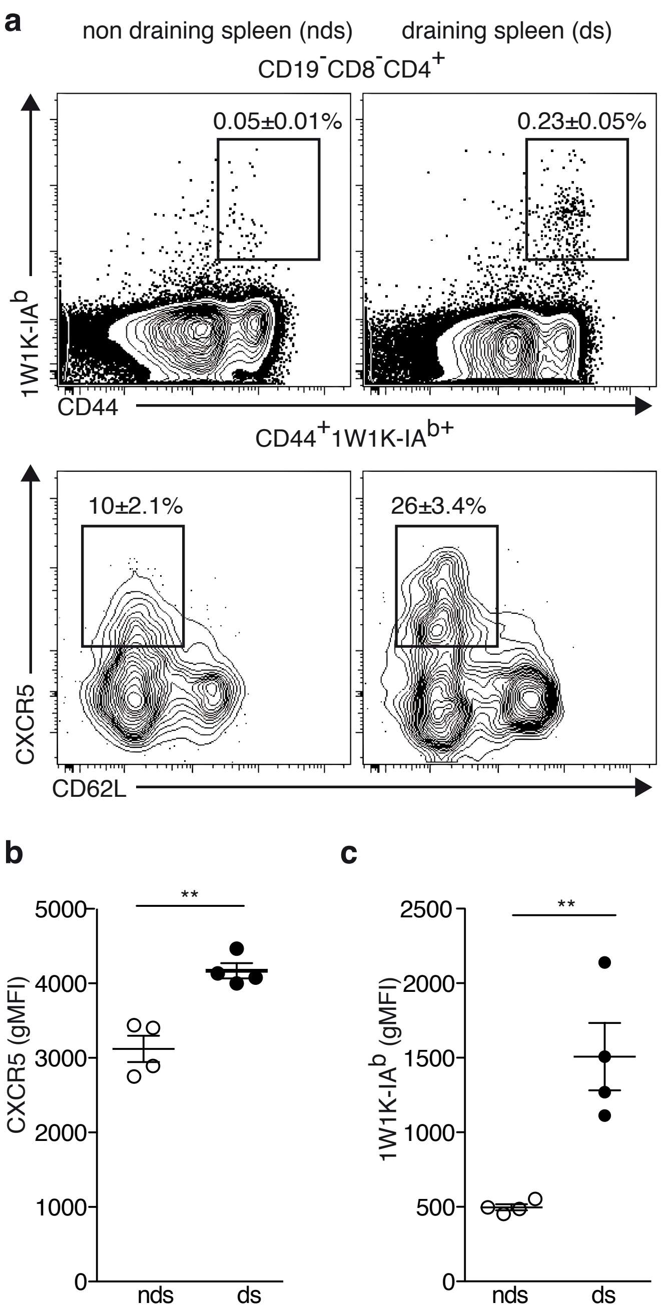

Supplementary Figure 3
Local and circulating 1W1K-specific memory Tfh in the spleen.
30 days after sc or ip immunization with 1W1K-OVA in SAS (Sigma Adjuvant System), non draining spleen from sc-immunized mice (nds) and draining spleen from ip-injected mice (ds) were analyzed for the detection of 1W1K-specific Th (1W1K-IAb^{+}CD44^{+}) (**a**, top) and 1W1K-specific Tfh (CXCR5^{+}CD62L^{-}) (**a**, bottom) (mean±SEM, n=5). (**b**) gMFI of CXCR5 staining and of 1W1K-IAb tetramer staining (**c**) at the surface of nds and ds 1W1K-specific mTfh at day 30 post-immunization.
Mann-Whitney test, *P<0.05; **P<0.01

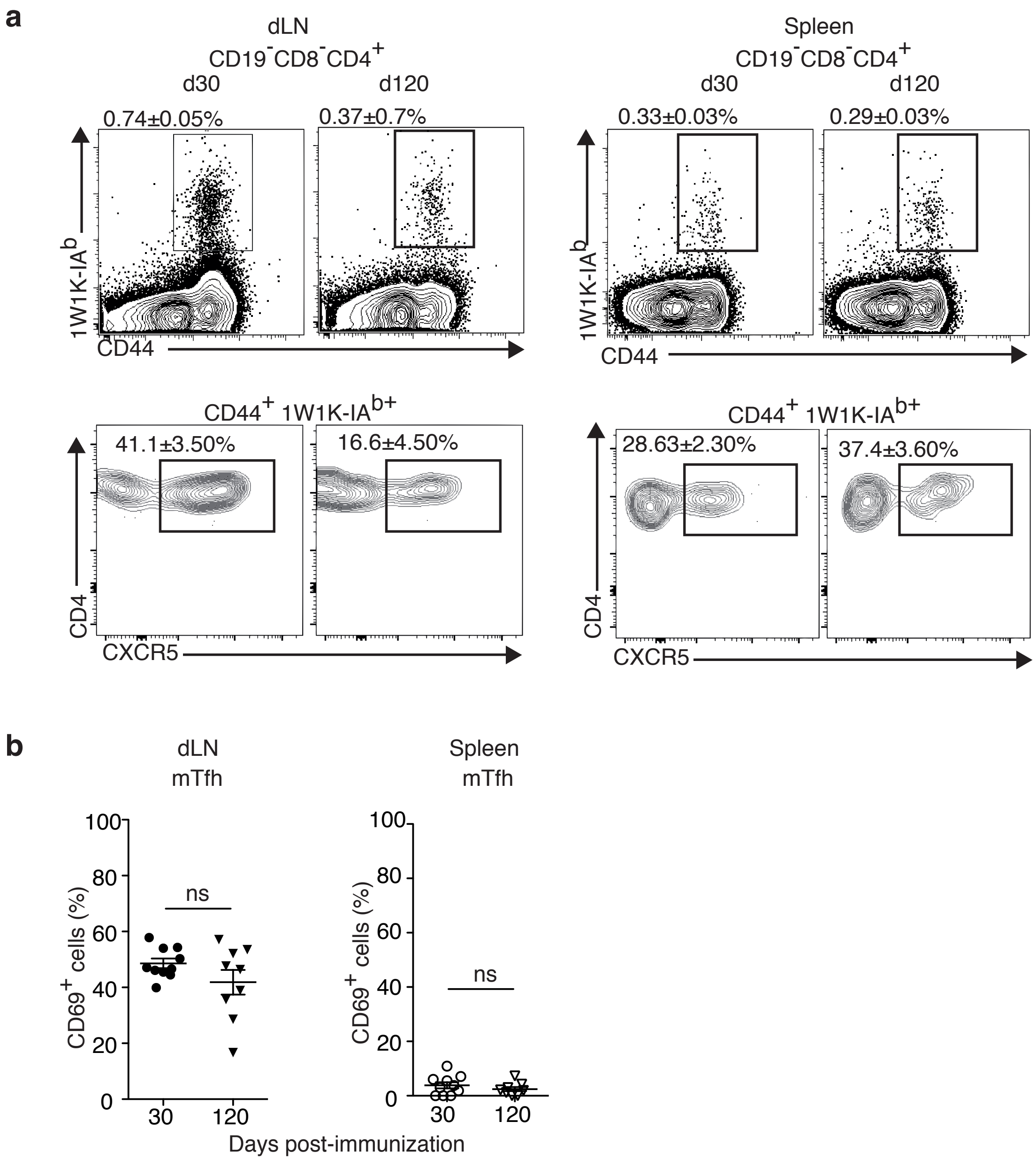

Supplementary Figure 4

Local and circulating 1W1K-specific memory Tfh cells 30 and 120 days post-immunization.

30 and 120 days after sc immunization with 1W1K-OVA in IFA+CpG, draining LN and non draining spleen were analyzed for the detection of 1W1K-specific Th (1W1K-IAb$^+$CD44$^+$) (**a**, top) and 1W1K-specific Tfh (CXCR5$^+$) (**a**, bottom) (mean±SEM, n=9).

(**b**) CD69 expression at the surface of dLN and spleen 1W1K-specific memory Tfh cells at day 30 and 120 post-immunization. Mann-Whitney test, ns, non significant

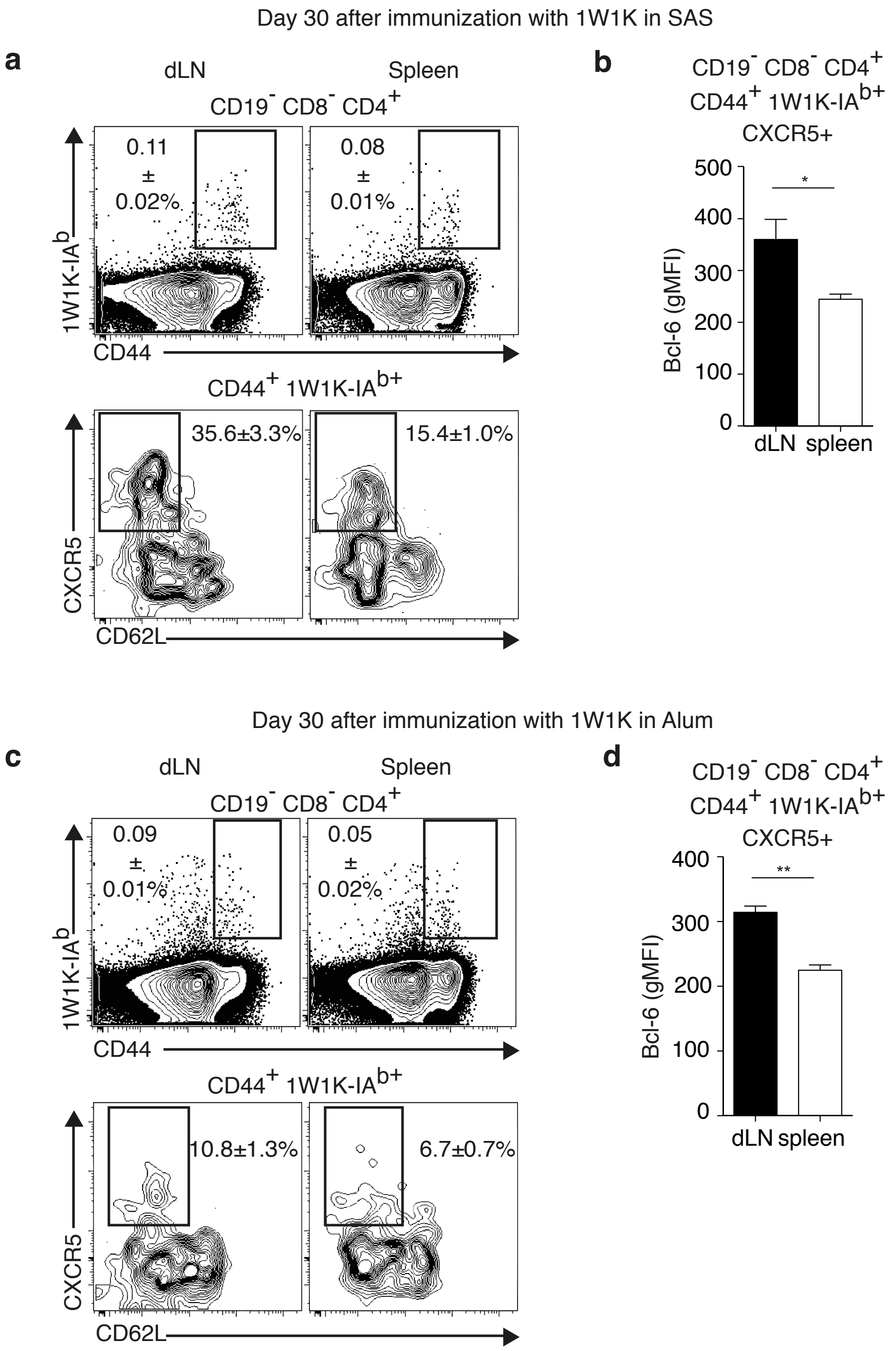

Supplementary Figure 5

Local and circulating 1W1K-specific memory Tfh after sc immunization with different adjuvants.

30 days after sc immunization with 1W1K-OVA in SAS (**a-b**) or in Alum (**c-d**), draining LN and spleen were analyzed for the detection of 1W1K-specific Th (1W1K-IAb^{+}CD44^{+}) (**a** and **c**, top) and 1W1K-specific Tfh (CXCR5^{+}CD62L^{-}) (**a** and **c**, bottom) (mean±SEM, n=5).

(**b** and **d**) gMFI of Bcl-6 staining of dLN and spleen 1W1K-specific memory Tfh at day 30 post-immunization. Mann-Whitney test, *P<0.05; **P<0.01

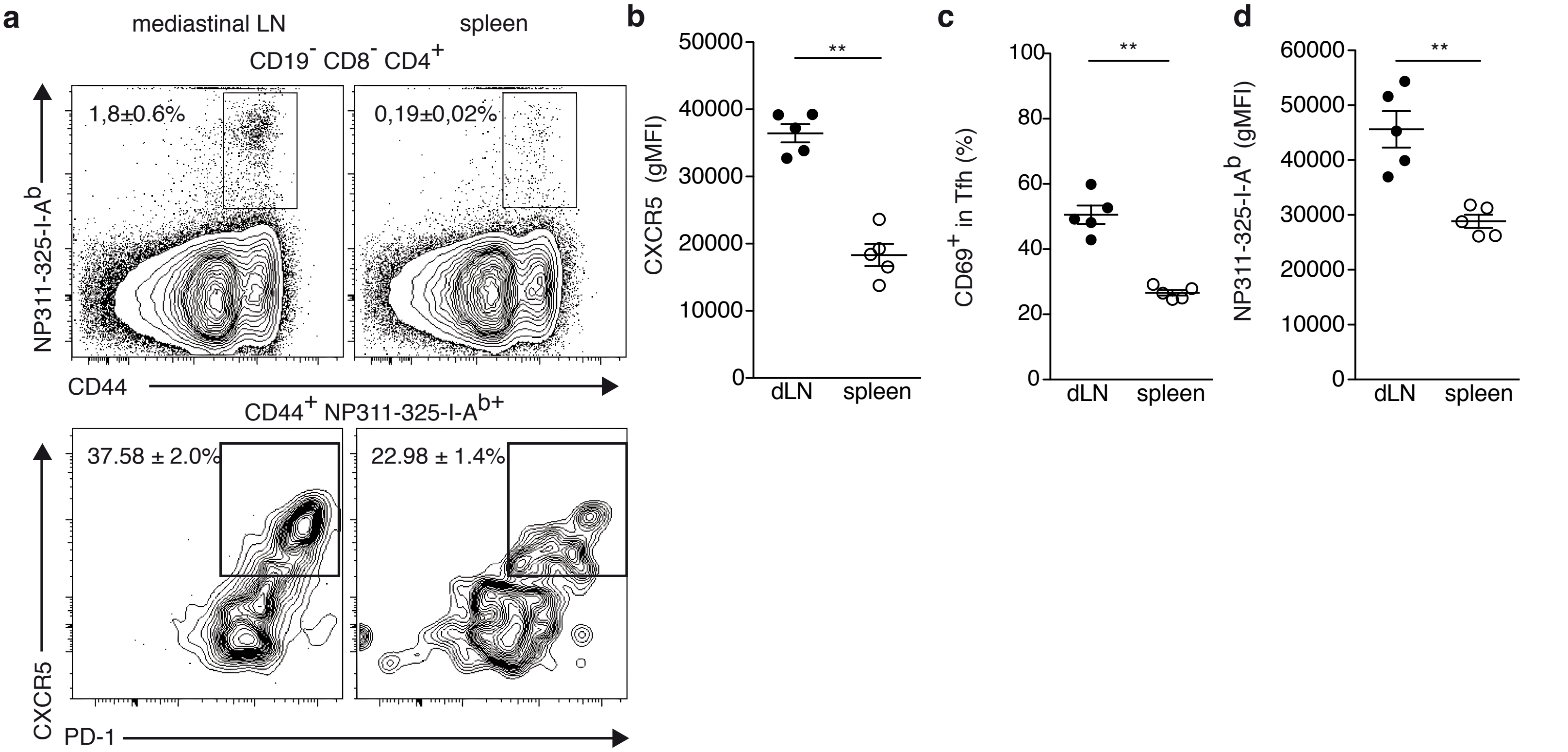

Supplementary Figure 6

Local and circulating NP311-325-specific memory Tfh cells after influenza infection.

45 days after intranasal infection with PR8 virus (MLD50 =1), mediastinal dLN and spleen were analyzed for the detection of NP311-325-specific Th (NP311-325-I-Ab^{+}CD44^{+}) (**a**, top) and Tfh (CXCR5^{+} PD-1^{+}) (**a**, bottom) (mean±SEM, n=5). gMFI of CXCR5 (**b**), frequency of CD69 (**c**) and gMFI of NP311-325-I-Ab^{+} tetramer staining (**d**) among NP311-325-specific Tfh cells.

Mann-Whitney test, *P<0.05; **P<0.01

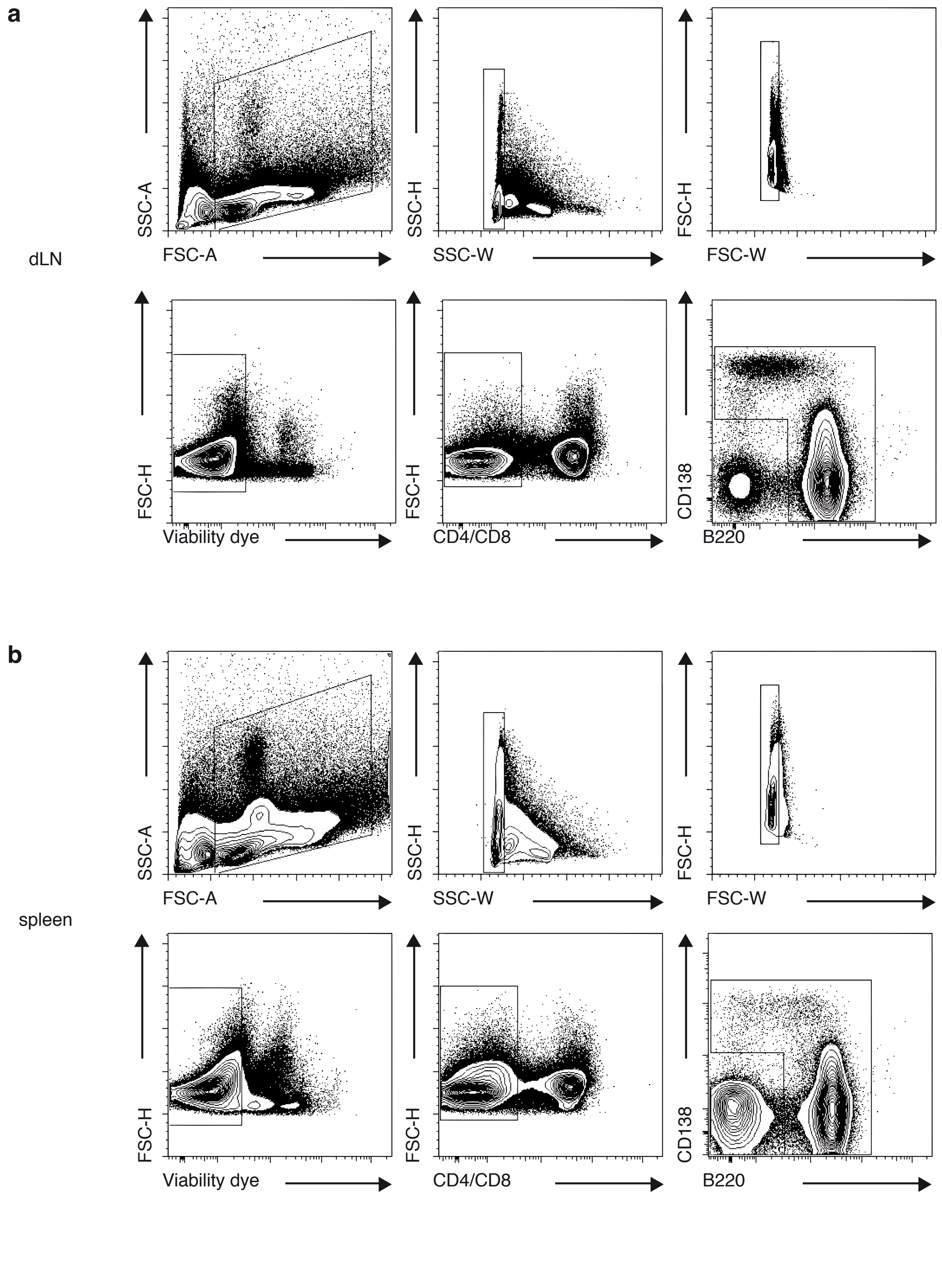

Supplementary Figure 7

Gating strategy to total B cells in dLN and in spleen 30 post-immunization

C57BL/6 mice were immunized sc with 100 μg NP-OVA in IFA+CpG.
30 days after, dLN and spleen from immunized mice were collected and were analyzed for the detection of total B cells by excluding dead cells, CD4/CD8$^+$ cells and by positively selecting C138/B220$^+$ cells.

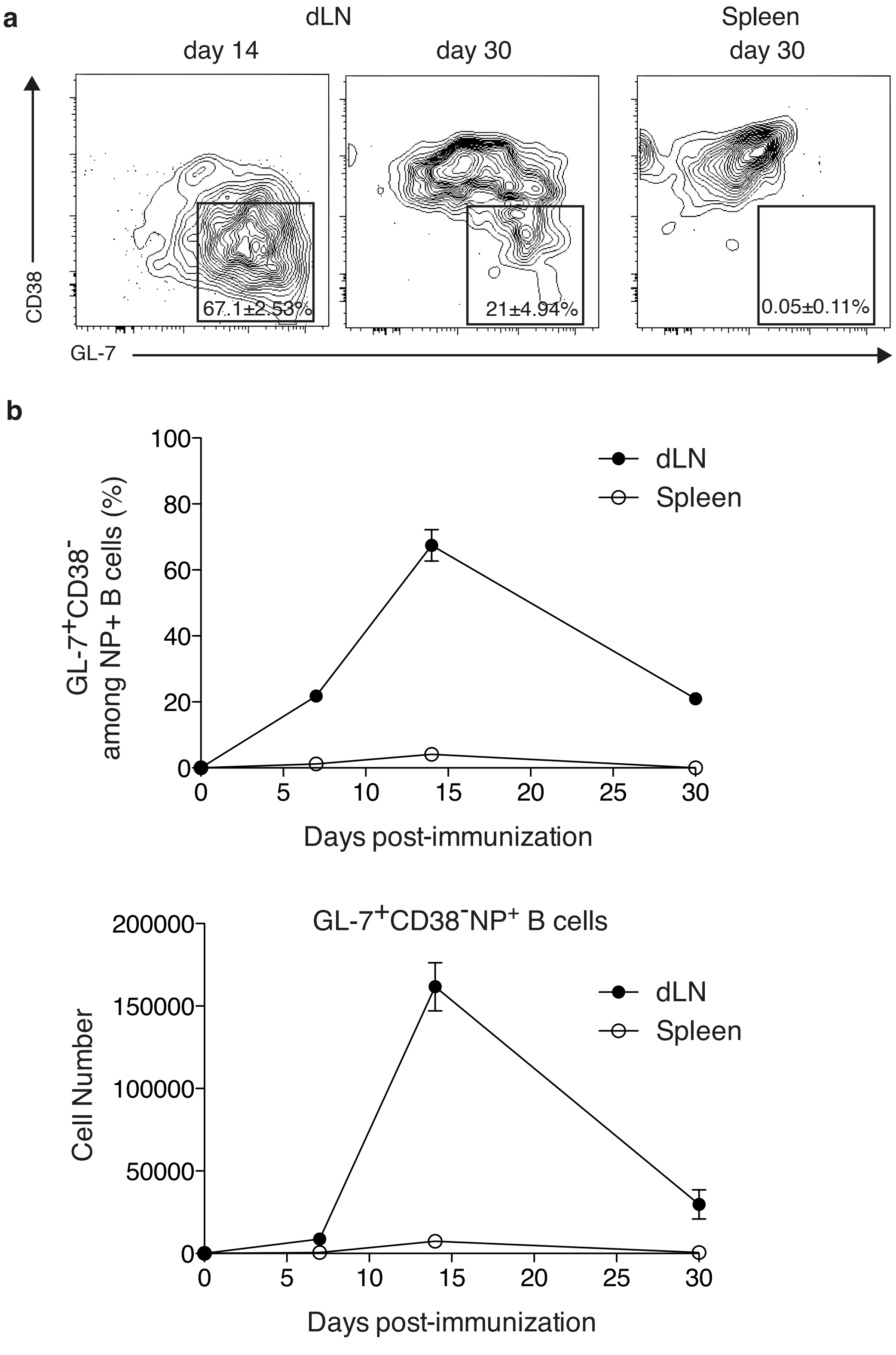

Supplementary Figure 8
Kinetics of NP^+ B cells
C57BL/6 mice were immunized sc with 100 μg NP-OVA in IFA+CpG. GL-7 and CD38 expression were monitored at the surface of NP^+ B cells. In a is presented the expression level of dLN and spleen cells day 14 and 30 post-immunization. In b are presented the kinetics of NP-specific $IgD^-CD38^-GL\text{-}7^+$ B cells after immunization (mean±SEM, n≥ 5/time point)

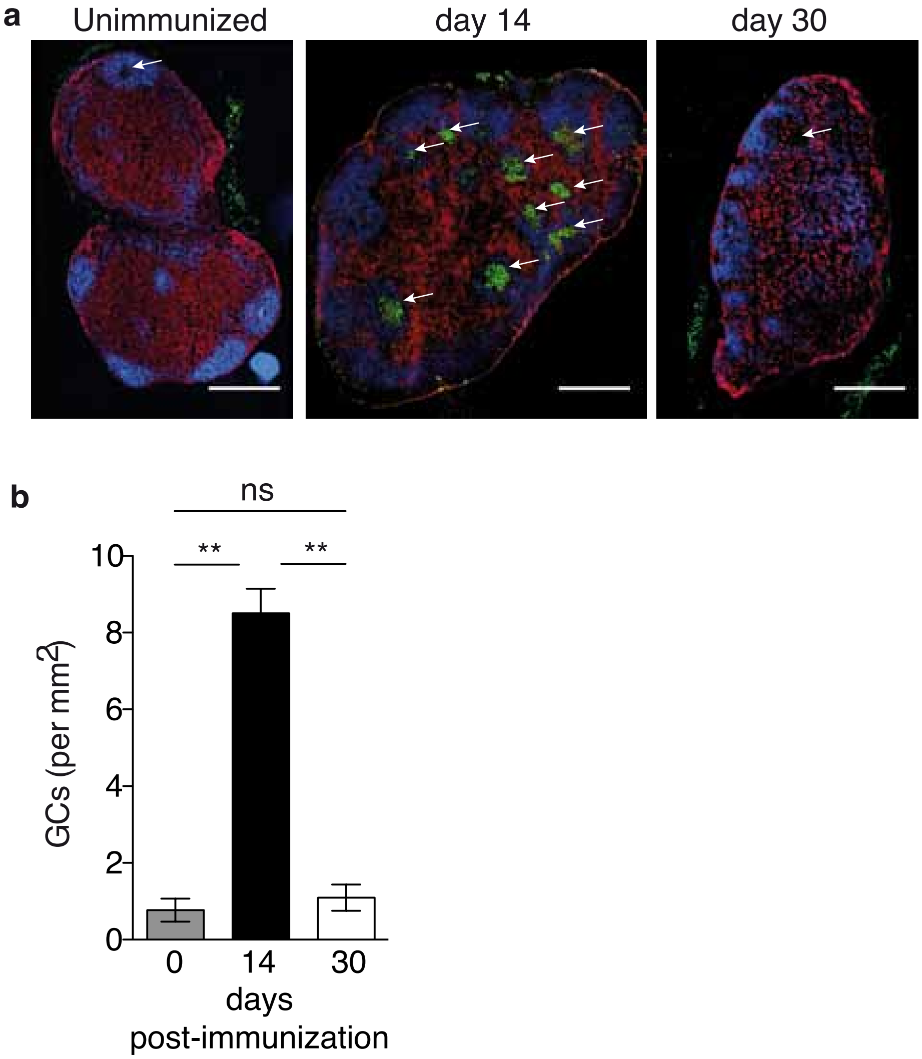

Supplementary Figure 9
Kinetics of GC reaction in dLN after 1W1K-OVA immunization
C57BL/6 mice were immunized sc with 1W1K-OVA in IFA+CpG. 14 and 30 days after, dLN from immunized mice or from naive mice were collected and confocal microscopy studies were performed using anti-IgD (blue), anti-GL-7 (green) and CD4 (red) mAb. LN were harvested into PLP buffer (0.05M phosphate buffer containing 0.2ml-lysine [pH 7.4], 2 mg/ml NaIO4, 10 mg/ml paraformaldehyde), fixed overnight and dehydrated in 30% sucrose prior to embedding in OCT freezing media (Sakura Fineteck). Frozen sections were cut on a CM1950 Cryostat. Sections were stained in PBS/1% BSA. Images were acquired on a Apotome ZEISS Inv. (scale bars; 200 μm). Number of GCs per mm2 in LN sections is depicted. n=8/conditions. mean+SEM. Mann-Whitney test, ns, non significant; **P<0.01

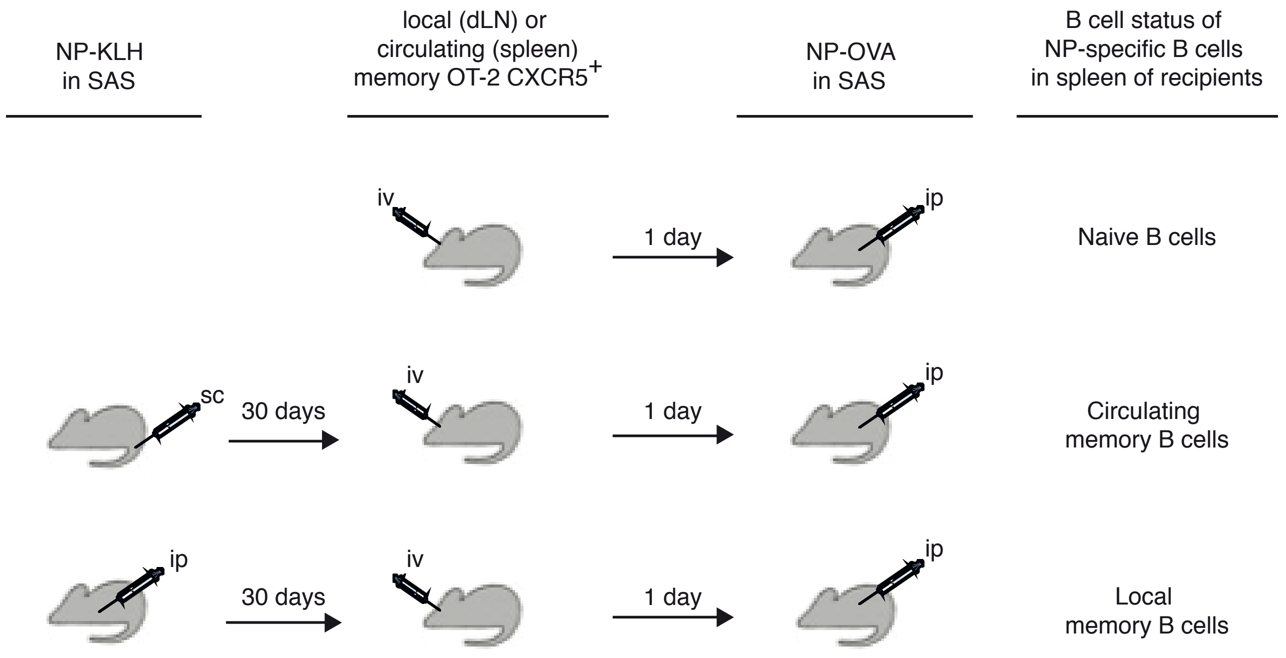

Supplementary Figure 10
Experimental scheme of *in vivo* transfer and immunization related to Figure 7
sc, subcutaneous; iv, intravenously; ip, intraperitonally.

a

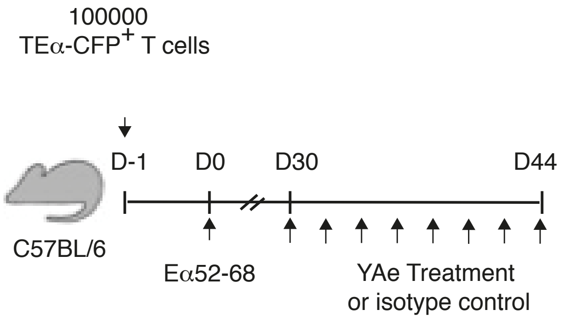

b

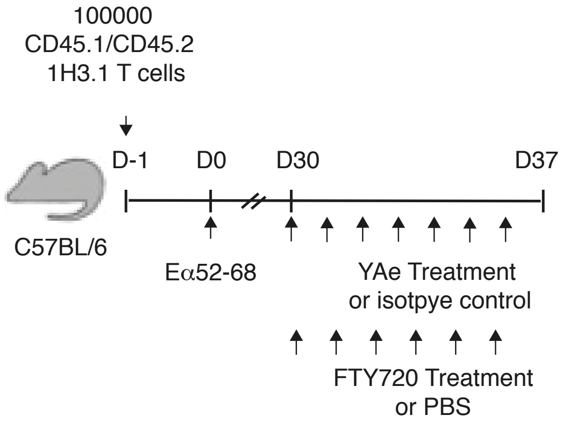

Supplementary Figure 11
Experimental schemes related to Figure 8

	Sequence
TCRVb1	CTGAATGCCCAGACAGCTCCAAGC
TCRVb2	TCACTGATACGGAGCTGAGGC
TCRVb3	CCTTGCAGCCTAGAAATTCAGT
TCRVb4	GCCTCAAGTCGCTTCCAACCTC
TCRVb5.1	CATTATGATAAAATGGAGAGAGAT
TCRVb5.2	AAGGTGGAGAGAGACAAAGGATTC
TCRVb5.3	AGAAAGGAAACCTGCCTGGTT
TCRVb6	CTCTCACTGTGACATCTGCCC
TCRVb7	TACAGGGTCTCACGGAAGAAGC
TCRVb8.1	CATTACTCATATGTCGCTGAC
TCRVb8.2	CATTATTCATATGGTGCTGGC
TCRVb8.3	TGCTGGCAACCTTCGAATAGGA
TCRVb9	TCTCTCTACATTGGCTCTGCAGGC
TCRVb10	ATCAAGTCTGTAGAGCCGGAGGA
TCRVb11	GCACTCAACTCTGAAGATCCAGAGC
TCRVb12	GATGGTGGGGCTTTCAAGGATC
TCRVb13	AGGCCTAAAGGAACTAACTCCCAC
TCRVb14	ACGACCAATTCATCCTAAGCAC
TCRVb15	CCCATCAGTCATCCCAACTTATCC
TCRVb16	CACTCTGAAAATCCAACCCAC
TCRVb17	AGTGTTCCTCGAACTCACAG
TCRVb18	CAGCCGGCCAAACCTAACATTCTC
TCRVb19	CTGCTAAGAAACCATGTACCA
TCRVb20	TCTGCAGCCTGGGAATCAGAA
TCRCb	GCAATCTCTGCTTTTGATGGCTC
Taqman Probe	FAM-AAATGTGACTCCACCCAAGGTCTCCTTGTT-TAMRA
TCRJb1.6	TCACAGTGAGCCGGGTGCCTGC
il2 forward	GTGCTCCTTGTCAACAGCG
il2 reverse	GGGGAGTTTCAGGTTCCTGTA
il21 forward	TCAGCTCCACAAGATGTAAAG
il21 reverse	GGGCCACGAGGTCAATGAT
ifng forward	CCCTCACACTCAGATCATCTT
ifng reverse	GCTACGACGTGGGCTACAG

Supplementary Table 1

Primer pairs used for quantitative PCR

All probe sequences are in the format 5’-sequence-3’
